# Advances and challenges in the application of prophylactic hyperthermic intraperitoneal chemotherapy for gastric cancer

**DOI:** 10.3389/fonc.2026.1679522

**Published:** 2026-02-10

**Authors:** Muyang Liu, Xiaojun Shen

**Affiliations:** 1 School of Medicine, Jiangsu University, Zhenjiang, Jiangsu, China; 2 Department of General Surgery, Kunshan First People’s Hospital, Kunshan, Jiangsu, China

**Keywords:** gastric cancer, overall survival, peritoneal metastasis, prophylactic hyperthermic intraperitoneal chemotherapy, recurrence

## Abstract

Gastric cancer is the fifth most common malignant tumor worldwide and the third leading cause of cancer-related deaths, posing a serious threat to global public health. Despite notable advancements in surgical techniques, systemic chemotherapy, targeted therapy, and immunotherapy in recent years, the overall prognosis for patients with locally advanced gastric cancer (AGC) remains suboptimal, primarily due to the high propensity for local and distant metastasis. Peritoneal metastasis (PM), in particular, has become one of the most formidable clinical challenges owing to its high incidence, inherent resistance to systemic chemotherapy, and extremely poor prognosis. To address this challenge, Prophylactic Hyperthermic Intraperitoneal Chemotherapy (P-HIPEC) has emerged as a focal point of research. This therapeutic modality aims to eradicate microscopic residual tumors and free cancer cells in the abdominal cavity following radical gastrectomy, thereby effectively reducing the risk of peritoneal metastasis and improving long-term patient survival. This review systematically examines the latest research progress of P-HIPEC in the treatment of gastric cancer, provides an in-depth analysis of its mechanism of action, indications, criteria for patient and drug selection, evidence of clinical efficacy, safety controversies, and challenges in standardization. It also looks forward to future research directions in this field, with the aim of providing a valuable reference for clinical practice and academic research.

## 1 Introduction

Gastric cancer constitutes a major global health burden and is the fifth most common malignant tumor and the third leading cause of cancer-related mortality worldwide (1). Despite multimodal advancements in surgical techniques, systemic chemotherapy, targeted therapy, and immunotherapy, the prognosis for patients with locally advanced gastric cancer (AGC) remains suboptimal (2). This is primarily attributed to a high rate of recurrence, with peritoneal metastasis (PM) emerging as a particularly challenging and frequent mode of treatment failure (3). The development of PM is associated with an inferior prognosis, as conventional systemic chemotherapy offers limited efficacy due to the restrictive nature of the plasma-peritoneal barrier (4). In response to this critical unmet need, Prophylactic Hyperthermic Intraperitoneal Chemotherapy (P-HIPEC) has been developed as a targeted locoregional strategy designed to eradicate residual microscopic disease and free-floating cancer cells within the peritoneal cavity (5).

This review aims to provide a comprehensive and systematic overview of the current research landscape of P-HIPEC for gastric cancer. Beyond merely compiling existing data, we critically evaluate how these findings directly translate to clinical decision-making, particularly in the era of modern systemic chemotherapy (e.g., FLOT regimen). We specifically address the heterogeneity in HIPEC protocols and provide actionable recommendations to aid oncology specialists in optimizing patient selection.

## 2 Gastric cancer and peritoneal metastasis

Peritoneal metastasis is one of the most common and detrimental forms of distant spread in advanced GC, and it is a primary cause of treatment failure and patient mortality.

### 2.1 Epidemiology

The peritoneum is one of the most common sites of metastasis after curative surgery for advanced gastric cancer, serving as the initial site of metastasis in approximately 40% to 60% of patients. For T3 and T4 stage gastric cancer, the incidence of peritoneal metastasis after radical surgery exceeds 50% (6). Once overt peritoneal metastasis occurs, the prognosis is extremely poor, with the median survival time dropping to 3 to 6 months and a 5-year survival rate of nearly 0% (7). Microscopic metastatic lesions and free cancer cells (FCCs) in the abdominal cavity are difficult to detect with conventional imaging techniques in the early stages. Furthermore, traditional systemic intravenous chemotherapy is largely ineffective because the peritoneal-plasma barrier prevents drugs from reaching therapeutic concentrations within the peritoneal cavity.

### 2.2 Molecular pathogenesis

**Figure 1:**
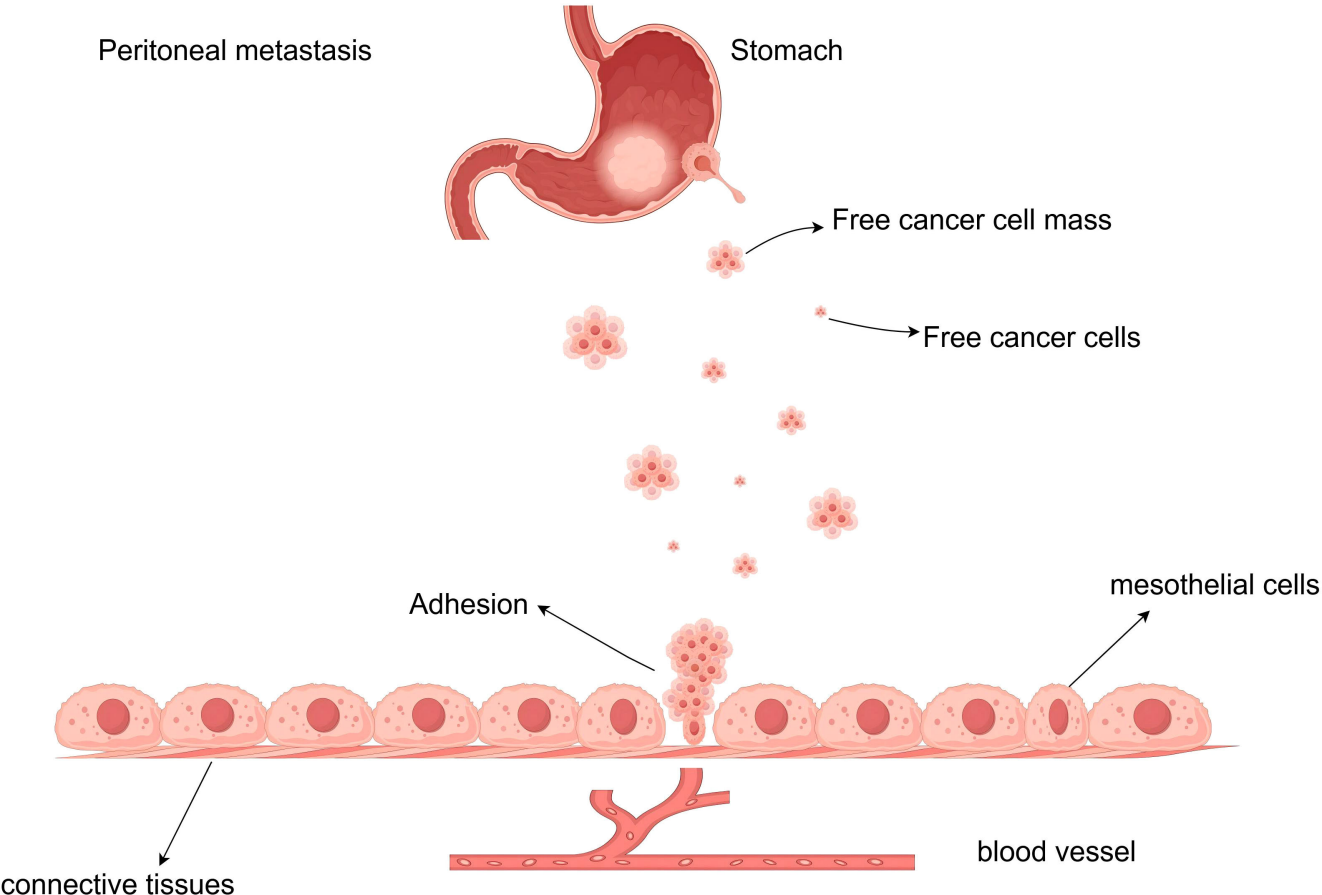
Process of peritoneal metastasis in gastric cancer. Conceptual cascade of peritoneal metastasis in gastric cancer and the perioperative window targeted by prophylactic HIPEC. Tumor serosal involvement facilitates exfoliation of free cancer cells (FCCs) into the peritoneal cavity, followed by survival, adhesion to injured/inflamed mesothelium, invasion, and outgrowth into macroscopic implants. P-HIPEC is delivered at curative-intent surgery to eradicate FCCs and microscopic disease before implantation becomes established. FCCs, free cancer cells; GC, gastric cancer; HIPEC, hyperthermic intraperitoneal chemotherapy; PM, peritoneal metastasis.

Peritoneal metastasis is a highly ordered, multi-step biological process governed by the “seed and soil” theory (**Figure 1**) (8). It begins with the detachment of cancer cells from the primary tumor, a process facilitated by Epithelial-Mesenchymal Transition (EMT). During EMT, downregulation of E-cadherin and activation of transcription factors (e.g., Snail, Twist, ZEB) confer migratory properties to gastric cancer cells (9–11). Once exfoliated, free cancer cells (FCCs) survive in the peritoneal cavity by aggregating into spheroids to resist anoikis and navigate toward the peritoneum via chemokine axes, such as CXCL12/CXCR4 (12, 13). Adhesion involves the interaction between cancer cells and the mesothelium. Tumor-derived TGF-β1 induces mesothelial-to-mesenchymal transition in peritoneal cells, exposing the basement membrane (14). Adhesion molecules such as integrin α3β1, CD44, and PCDHB9 then anchor cancer cells to the submesothelial matrix (15–17). Finally, tumor cells secrete matrix metalloproteinases (MMPs) to degrade the extracellular matrix and Vascular Endothelial Growth Factor (VEGF) to induce angiogenesis, establishing macroscopc nodules (17, 18).

The existence of the plasma-peritoneal barrier makes it difficult for traditional systemic intravenous chemotherapy to achieve effective cytotoxic concentrations within the peritoneal cavity. Therefore, the development of local therapeutic strategies that act directly on the peritoneum has become an urgent need.

## 3 Mechanism of action of HIPEC

**Figure 2:**
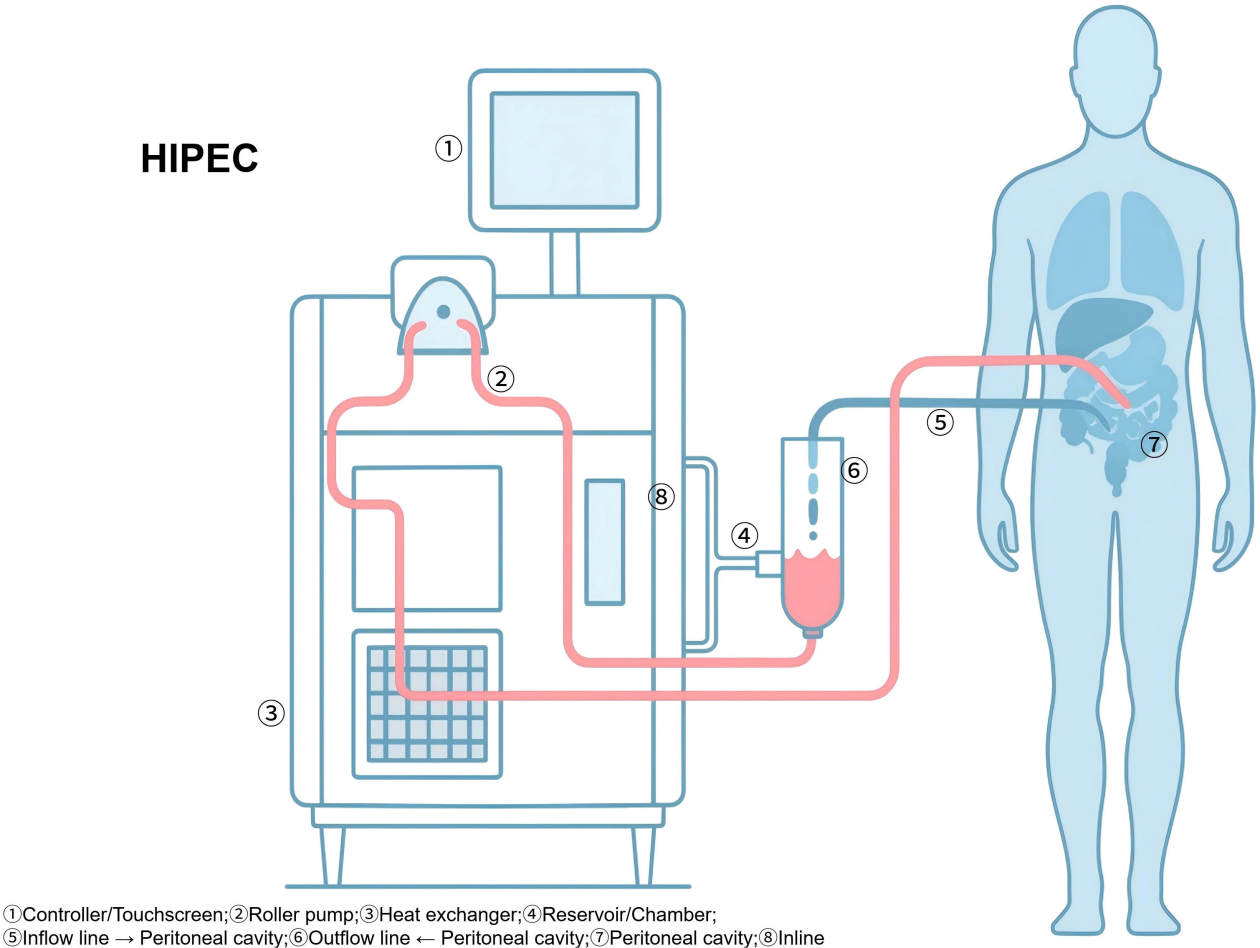
Schematic of hyperthermic intraperitoneal chemotherapy (HIPEC). Schematic overview of intraoperative HIPEC delivery. Representative elements include inflow/outflow catheter placement, perfusion circuit with heating/exchanger, target intraperitoneal temperature monitoring, and differences between open (Coliseum) and closed techniques. HIPEC, hyperthermic intraperitoneal chemotherapy.

At least one inflow catheter and one to two outflow catheters are positioned intraperitoneally, along with temperature probes placed in different abdominal quadrants. The perfusion device is primed with isotonic carrier solution and gradually heated until both the perfusion circuit and intraperitoneal cavity temperatures stabilize within the range of 41–43°C. Chemotherapeutic agents are then administered based on the patient’s body surface area or a predetermined protocol. A suitable perfusion flow rate is set, commonly between 0.8 and 1.5 L/min, adjusted according to device characteristics and intraperitoneal capacity, and the chemotherapy is circulated for a duration of 60–90 minutes. Throughout the perfusion, patient positioning is periodically adjusted and gentle abdominal manipulation is performed to facilitate uniform distribution of the therapeutic solution. Continuous monitoring of inflow and outflow temperatures, as well as multipoint intraperitoneal temperatures, is conducted to maintain the desired therapeutic range. Upon completion, the peritoneal cavity is thoroughly rinsed with warmed saline, residual perfusate is collected and properly disposed of according to standard guidelines, drainage tubes may be retained as clinically indicated, and surgical closure is performed (**Figure 2**).

The clinical efficacy of prophylactic HIPEC stems from the synergistic effects of multiple actions, including hyperthermia (HT), high-dose regional chemotherapy, mechanical flushing, and immunomodulation. This technology is designed to completely eradicate the residual microscopic tumor burden within the peritoneal cavity.

### 3.1 Hyperthermia

**Figure 3:**
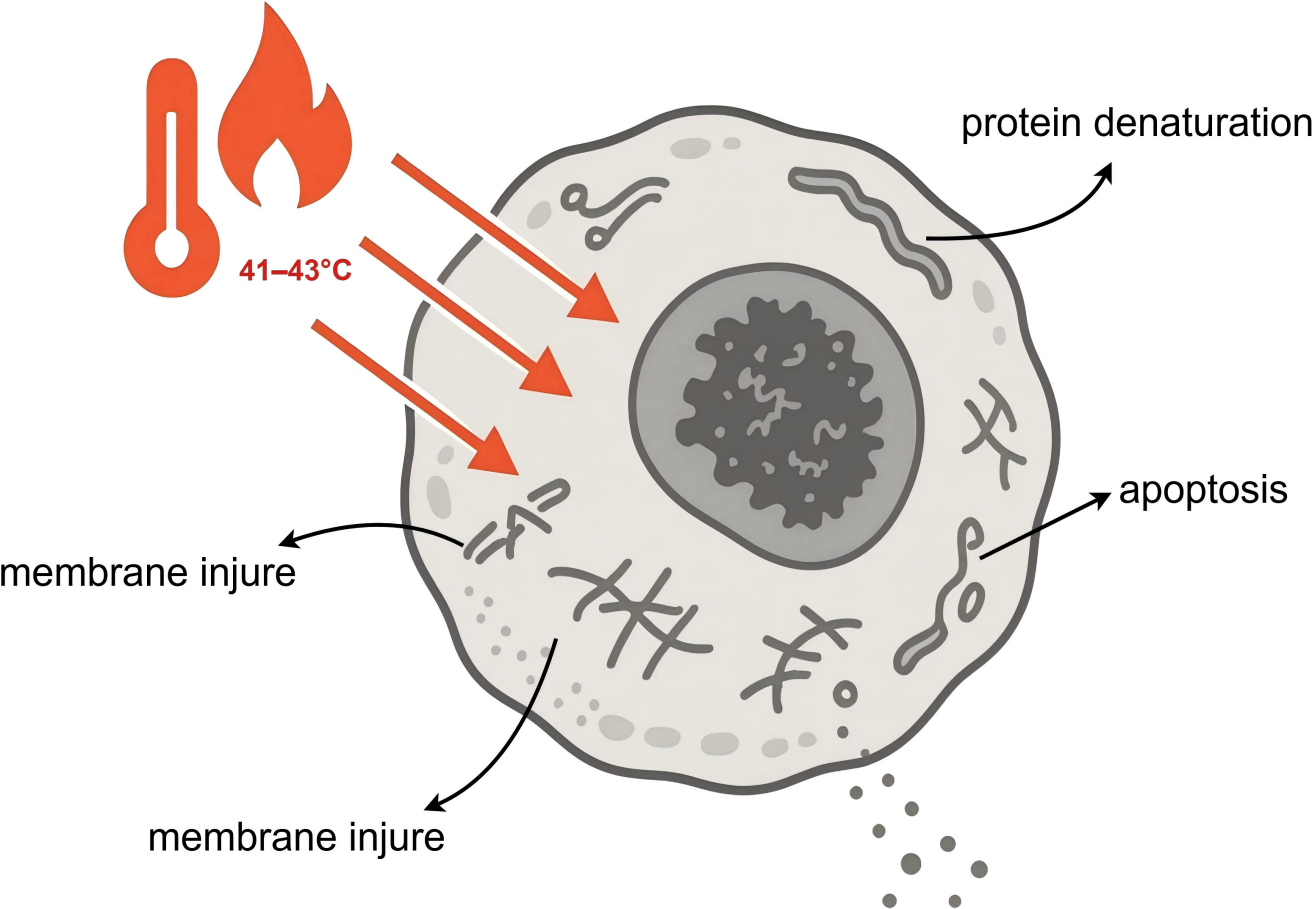
Direct cytotoxic effects of hyperthermia (41-43°C) on tumor cells. Clinically relevant effects of hyperthermia combined with intraperitoneal chemotherapy. Hyperthermia increases local cytotoxicity, improves peritoneal surface drug penetration, and can impair DNA damage repair, thereby sensitizing tumor cells to chemotherapy. The magnitude of effect depends on protocol parameters (temperature stability, exposure duration, and drug regimen). DDR, DNA damage repair; HIPEC, hyperthermic intraperitoneal chemotherapy.

**Figure 4:**
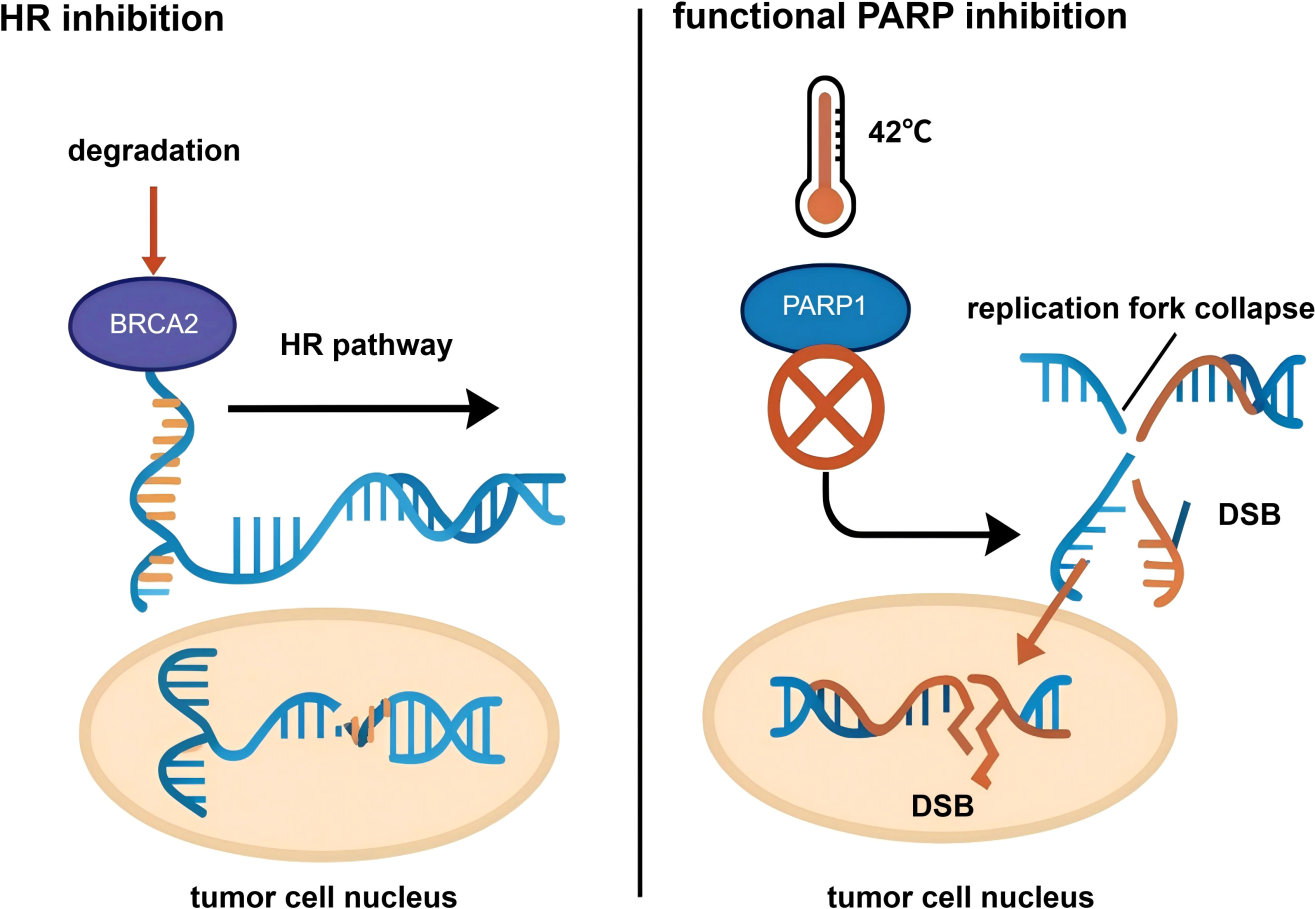
Hyperthermia-induced HR and PARP inhibition. Proposed interaction between hyperthermia-induced impairment of homologous recombination and PARP inhibition as a rationale for future combination strategies. Hyperthermia may transiently reduce homologous recombination capacity, potentially creating a vulnerability that could be exploited by PARP inhibitors in selected contexts. HR, homologous recombination; PARP, poly(ADP-ribose) polymerase.

Hyperthermia is the core element that distinguishes HIPEC from normothermic intraperitoneal chemotherapy (NIPEC), and it possesses multi-level anti-tumor effects. ① Direct cytotoxicity: Numerous experiments have confirmed that tumor cells are significantly more sensitive to heat than normal tissue cells due to their disorganized vascular structure, poor blood supply, and acidic microenvironment (19, 20). Continuous exposure to a temperature of 43°C for 1 hour can cause irreversible damage to tumor cells, leading to apoptosis or necrosis, whereas normal tissue cells can tolerate temperatures up to 47°C. Thermal effects can directly damage the cell membrane and organelles (such as lysosomes and mitochondria) of cancer cells and interfere with their energy metabolism(**Figure 3**) (21). ② Enhancement of chemotherapeutic drug efficacy: There is a synergistic effect between hyperthermia and chemotherapy drugs. High temperatures increase the fluidity and permeability of the tumor cell membrane, promoting more effective entry of chemotherapy drugs into the cancer cells. Additionally, hyperthermia can alter blood perfusion in tumor tissues, increasing drug accumulation. Critically, hyperthermia can interfere with the self-repair capabilities of tumor cells at the molecular level (22–24). ③ Inhibition of DNA Damage Repair (DDR): Many chemotherapy drugs (e.g., platinum agents, mitomycin C) kill cancer cells by causing DNA damage. However, cancer cells possess a complex DNA Damage Repair (DDR) system to counteract this damage (25). Research shows that hyperthermia can effectively inhibit DDR, making cancer cells more vulnerable to chemotherapeutic attack: (i) Inhibition of Homologous Recombination (HR) repair: HR is a key pathway for repairing DNA double-strand breaks (DSBs) (26). Hyperthermia has been shown to promote the degradation of the key repair protein BRCA2, thereby significantly inhibiting the activity of the HR pathway (27) (**Figure 4**). (ii) Functional PARP inhibition: A recent groundbreaking study revealed a deeper synergistic mechanism of hyperthermia. Poly(ADP-ribose) polymerase 1 (PARP1) is a key sensor and signaling molecule in DDR. When chemotherapy drugs cause DNA damage, PARP1 is activated (28). PARP1 catalyzes a modification called PARylation to recruit repair proteins and pause the cell cycle, allowing time for DNA repair. The study found that hyperthermia at 42°C can efficiently inhibit the enzymatic activity of PARP1, with an effect comparable to that of pharmacological PARP inhibitors (PARPi) (29). By inhibiting PARP1, hyperthermia prevents the normal replication fork slowing and repair processes after DNA damage, leading to a dramatic increase in replication stress under continuous chemotherapy. This results in the formation of a large number of irreparable, lethal DSBs, thereby greatly enhancing the cytotoxic effect of chemotherapy. This finding provides a solid theoretical basis for prioritizing DNA-damaging agents in HIPEC regimens (**Figure 4**).

### 3.2 Pharmacokinetics

The cornerstone of HIPEC’s theoretical basis lies in its unique pharmacokinetic advantage: utilizing and overcoming the plasma-peritoneal barrier. This semipermeable membrane structure hinders the absorption of large-molecule, hydrophilic chemotherapy drugs from the peritoneal cavity into the systemic circulation (30). Consequently, direct intraperitoneal administration can achieve extremely high drug concentrations within the peritoneal cavity—potentially tens of times higher than those achievable with safe systemic administration—while systemic drug exposure and toxic side effects remain relatively low (31–33). This high-concentration local chemotherapy environment is crucial for eradicating free cancer cells and micrometastases in the abdominal cavity, as it can overcome the relative resistance of tumor cells to conventional doses of chemotherapy. However, this advantage is accompanied by an inherent limitation: the penetration depth of most chemotherapy drugs into peritoneal tissue is very limited, typically only 2–5 mm (24). This fact underscores why HIPEC must be combined with thorough surgical intervention. The core principle established by pioneers like Sugarbaker is that any visible tumor nodule larger than this penetration depth must be removed through cytoreductive surgery (CRS). The role of HIPEC is to treat the “residual” disease that is not visible to the naked eye. Without thorough CRS as a prerequisite, the efficacy of HIPEC is greatly diminished (30).

### 3.3 Mechanical flushing and immunomodulation

① Physical removal: During the HIPEC procedure, a large volume (typically 4–6 liters) of perfusate circulates at high speed within the peritoneal cavity. This continuous mechanical flushing physically removes and dilutes suspended and unattached FCCs, reducing the probability of their implantation (30). ② Immune activation effect: Hyperthermia itself is a stressor that can induce tumor cells to express Heat Shock Proteins (HSPs). When released extracellularly, these HSPs can act as a “danger signal,” recognized and captured by antigen-presenting cells such as Dendritic Cells (DCs). This promotes DC maturation and enhances their ability to present tumor-associated antigens, ultimately activating a specific T-cell immune response against the tumor and producing a long-term anti-tumor effect (34).

## 4 Indications and patient selection for P-HIPEC

P-HIPEC is not suitable for all gastric cancer patients; strict patient selection is a prerequisite for ensuring its efficacy and safety. The core principle for applying P-HIPEC is for patient subgroups who have no macroscopic peritoneal metastasis (Peritoneal Carcinomatosis Index, PCI = 0) but are judged to have a high risk of peritoneal metastasis based on clinicopathological features (3). Based on multiple clinical studies, systematic reviews, and expert consensus, the currently recognized high-risk factors for peritoneal metastasis in gastric cancer include: ① T stage: Tumor invasion to the subserosa (pT3) or perforation of the serosa (pT4a) is the most important criterion for selecting patients for P-HIPEC (35). Notably, tumor invasion into adjacent structures (pT4b) represents the highest risk for exfoliation and is considered a strong indication for P-HIPEC in many protocols (36). ② N stage: Positive lymph node metastasis (N+) is also a recognized high-risk factor, indicating a stronger invasive and metastatic capacity of the tumor. ③ Peritoneal Cytology: In the absence of macroscopic peritoneal nodules (P0), the detection of cancer cells in peritoneal washings (CY1) is a sign of extremely poor prognosis, with a 5-year survival rate comparable to that of patients with macroscopic metastases. Therefore, a CY1 status is considered by many scholars as an indication for prophylactic or early therapeutic HIPEC (37). It is noteworthy, however, that some strictly defined clinical trials, aiming to assess purely prophylactic effects, have excluded CY1 patients, considering them to have M1 stage metastasis (37, 38). This ambiguity in the definition and stratification of CY1 patients is a significant reason why results from different studies are difficult to compare directly. ④ Histological type: Specific histological types are closely related to the risk of peritoneal metastasis. According to the Lauren classification, diffuse-type gastric cancer is more prone to peritoneal dissemination than intestinal-type. Patients histologically diagnosed with signet ring cell carcinoma also have a significantly increased risk of peritoneal metastasis (39). ⑤ Other high-risk factors: Borrmann type IV (linitis plastica), tumor perforation or intraoperative rupture, and lymphovascular invasion are also considered high-risk markers for peritoneal metastasis (40). Furthermore, the “Expert Consensus on the Clinical Application of Hyperthermic Intraperitoneal Chemotherapy Technology (2016 Edition)” from China and its subsequent updates provide clear guidance for the clinical application of P-HIPEC (41). The consensus details the above high-risk factors as indications and specifies contraindications, including: ① Absolute contraindications: Confirmed distant organ metastases (e.g., liver, lung, bone), extensive retroperitoneal lymph node metastasis. ② Relative contraindications: Poor general condition of the patient (e.g., ECOG score >1 or KPS score <70), advanced age (usually >75 years) or very young age (<20 years), severe dysfunction of vital organs such as the heart, lungs, liver, or kidneys, rendering the patient unable to tolerate prolonged surgery and chemotherapy.

Patient selection for P-HIPEC is a process of comprehensive evaluation by a multidisciplinary team (MDT), which requires combining findings from preoperative imaging, endoscopic ultrasound, pathology, and intraoperative exploration, and conducting a thorough assessment of the patient’s risks and potential benefits.

While the “Expert Consensus on the Clinical Application of Hyperthermic Intraperitoneal Chemotherapy Technology” from China provides clear guidance, international perspectives vary. The PSOGI (Peritoneal Surface Oncology Group International) and recent European guidelines also advocate for P-HIPEC in high-risk groups, particularly T4 tumors, though criteria regarding CY1 status remain a subject of ongoing standardization (42–44).

## 5 Drug and regimen selection for P-HIPEC

The development of a P-HIPEC regimen involves multiple aspects, including the choice of chemotherapeutic agents, setting of technical parameters, and timing of implementation. However, there is currently no globally unified, standardized protocol in this field. The significant variations in regimens used by different research centers are a major cause of heterogeneity in study results. The selection of drugs for HIPEC should follow several basic principles: ① Efficacy against gastric cancer cells: The drug itself must be cytotoxic to gastric cancer. ② Hyperthermic sensitization effect: The drug’s anti-tumor activity should be enhanced at high temperatures. ③ Pharmacokinetic advantages: The drug should have characteristics such as a large molecular weight, good water solubility, and low absorption from the peritoneum, allowing it to be confined within the peritoneal cavity. ④ Good safety profile: The drug should have low peritoneal irritability and be unlikely to cause severe chemical peritonitis or intestinal adhesions.

The drugs more commonly used in clinical practice are: ① Mitomycin C (MMC): As an alkylating agent, MMC is one of the oldest and most widely used drugs in the history of HIPEC. It has a large molecular weight and a clear hyperthermic sensitization effect. Many early key studies used MMC monotherapy for P-HIPEC (45). ② Platinum Agents: Cisplatin (CDDP) and Oxaliplatin have become mainstream choices in modern HIPEC regimens due to their potent DNA cross-linking damage and proven significant hyperthermic sensitization effects. These drugs can be used alone or in combination with other agents (46). For example, the ongoing large-scale European Phase III clinical trial (GASTRICHIP) uses oxaliplatin (47). ③ Taxanes: Paclitaxel (PTX) and Docetaxel (DOC) are also used in HIPEC research due to their unique anti-tumor mechanisms and favorable intraperitoneal pharmacokinetic properties. These drugs can also be combined with platinum agents to achieve a stronger synergistic effect (48, 49). ④ Combination regimens: To further improve efficacy, some research centers have begun to explore combination drug regimens, such as cisplatin combined with docetaxel (49), or more complex three-drug combinations (e.g., MMC+CDDP+5-FU). However, the safety and efficacy of these combination regimens still require confirmation from more high-quality clinical trials.

In addition to drug selection, the specific technical parameters of HIPEC also vary: ① Perfusion technique: This is mainly divided into the open (Coliseum technique) and closed methods. The open technique is performed under direct vision, which theoretically allows for more uniform drug distribution but has disadvantages such as aerosolization of chemotherapy drugs, increased exposure risk for the surgical team, and rapid heat loss. The closed technique is performed after closing the abdominal wall, offering higher safety but potentially leading to uneven drug distribution. ② Temperature: Most study protocols set the target intraperitoneal temperature at 42-43°C, a range that balances the therapeutic effect of hyperthermia with the risk of tissue damage. ③ Duration: The perfusion duration is typically 60 to 90 minutes, with the specific time depending on the properties of the selected drug and the study protocol design. ④ Timing: The vast majority of P-HIPEC procedures are performed as a single session after the completion of radical gastrectomy, either before or after gastrointestinal reconstruction. However, a few studies are exploring a model of early postoperative (e.g., 1–2 days post-op) fractionated perfusion through a pre-placed catheter, the pros and cons of which are yet to be determined. The following table summarizes the P-HIPEC regimens used in some key clinical trials to visually demonstrate their diversity (**Table 1**).

**Table 1:** Prophylactic HIPEC regimens in key clinical trials.

Trials	Patient population	HIPEC drugs	Temperature(°C)	Duration(mins)	Timing	Systemic chemotherapy	Source
GASTRICHIP	Gastrc cancer T3 or T4 and/or N+and/or with positive cytology(Cy+)	Oxaliplatin 250mg/m^2^ into 2 Liters/m^2^ of G5% Folinic Ac 20mg/m^2^ IVD 5-FU 400 mg/m² IVD	42-43	30	Intraoperative	Perioperative Chemotherapy	(47)
PREVENT(FLOT9)	localized and locally advanced diffuse or mixed type adenocarcinoma of the stomach and type II/III GEJ (i.e. ≥cT3 any N or any T N-positive) with laparoscopic exclusion of peritoneal seeding and radiological exclusion of other distant metastases	Cisplatin 75mg/m^2^	42	90	Intraoperative	Perioperative FLOT	(50)
Koga et al.	macroscopic serosal invasion and no macroscopic peritoneal metastasis	Mitomycin C 8-10mg/L	40-42	50-60	Intraoperative	No standardized regimen	(45)
CHIMERA	advanced gastric cancer at high risk of peritoneal metastases	Irinotecan 300mg/m^2^	42	45	Intraoperative	Perioperative FLOT	(51)

Representative randomized trials investigating prophylactic or early-therapeutic HIPEC strategies in gastric cancer. Trial characteristics (population, systemic therapy backbone, HIPEC drug/dose/temperature/duration, timing, and primary endpoint) were extracted from peer-reviewed protocol papers and/or registry records as cited in the “Source” column. DFS, disease-free survival; GC, gastric cancer; HIPEC, hyperthermic intraperitoneal chemotherapy; OS, overall survival; PM, peritoneal metastasis.

This heterogeneity in regimens is a major challenge facing research in the P-HIPEC field. It directly affects the comparability of results from different studies and hinders the formation of unified clinical practice guidelines. Future research urgently needs to determine the optimal combination of drugs, dosages, and technical parameters through well-designed head-to-head comparative trials.

## 6 Clinical efficacy analysis of P-HIPEC

The clinical efficacy of P-HIPEC is primarily measured by its impact on peritoneal metastasis rate, disease-free survival (DFS), and overall survival (OS). Existing evidence shows varying degrees of consistency and controversy across these different endpoints.

To improve transparency and avoid mixing fundamentally different intents, we now clearly distinguish prophylactic HIPEC (P-HIPEC; high-risk P0 disease at curative gastrectomy) from therapeutic CRS+HIPEC (macroscopic peritoneal carcinomatosis/metastasis) and anchor each PM/DFS/OS statement to specific RCTs and the most recent meta-analyses. In the prophylactic setting, randomized-trial meta-analyses consistently suggest reduced peritoneal failure: Sun et al. reported fewer peritoneal dissemination events (RR 0.69, 95% CI 0.52–0.93) and fewer peritoneal recurrence-related deaths (RR 0.45, 95% CI 0.27–0.82), but with substantial heterogeneity for the latter endpoint (I²=62%), highlighting the impact of patient selection, regimens, and technique. In the same analysis, OS showed low heterogeneity (I²=0%) (52). Method-specific pooling further supports regimen/technique dependence: Huang et al. reported a favorable 5-year recurrence signal for intraoperative hyperthermic IPC (HIIC) (RR 0.47, 95% CI 0.39–0.56) with moderate heterogeneity (I²=39%), and Yan et al. demonstrated that heterogeneity differs by IPC category (e.g., NIIC I²=48.9% vs HIIC I²=27.7%), arguing against a single “one-size-fits-all” pooled conclusion (53).

In contrast, the therapeutic setting should be reported separately: the phase III RCT by Yang et al. in gastric cancer peritoneal carcinomatosis showed improved median survival with CRS+HIPEC versus CRS alone (11.0 vs 6.5 months; p=0.046), underscoring that established PM populations and outcomes are not directly comparable to prophylaxis (54). Finally, we explicitly note that much prophylactic randomized evidence is derived from Asian, largely pre-FLOT eras, limiting Western generalizability; ongoing European phase III programs (e.g., GASTRICHIP and PREVENT) are designed to test P-HIPEC within contemporary staging and perioperative chemotherapy frameworks and will be critical to clarify external validity.

### 6.1 Peritoneal metastasis rate

P-HIPEC has demonstrated its most definitive clinical benefit in reducing the risk of peritoneal metastasis. The vast majority of evidence, from early retrospective studies to recent randomized controlled trials (RCTs) and meta-analyses, consistently indicates that the addition of P-HIPEC to surgery or surgery combined with systemic chemotherapy significantly reduces the incidence of postoperative peritoneal metastasis. Multiple meta-analyses report odds ratios (OR) concentrated in the range of 0.22 to 0.24, which means P-HIPEC can reduce the risk of peritoneal metastasis by approximately 75% to 78%, a clinically significant finding (52, 53, 55, 56). This result strongly supports the theoretical basis of P-HIPEC as a locoregional preventive measure.

### 6.2 Disease-free survival

Given its effectiveness in controlling peritoneal metastasis, the improvement in disease-free survival with P-HIPEC has also been widely confirmed. Multiple studies, including some high-quality propensity score matching (PSM) analyses and meta-analyses, have reported that DFS is significantly better in the P-HIPEC group compared to the control group. For instance, a recent retrospective study using PSM analysis found that the median DFS in the P-HIPEC group was 49.9 months, compared to only 26.9 months in the control group, with a hazard ratio (HR) of 0.569 (p=0.013), a statistically significant difference (6). This indicates that P-HIPEC not only prevents metastasis at a specific site but also effectively prolongs the time from surgery to the first recurrence.

### 6.3 Overall survival

Whether P-HIPEC improves overall survival remains the central and unresolved controversy. The literature can be interpreted in two contrasting directions: (1) Evidence suggesting an OS benefit: Several meta-analyses—largely driven by Asian cohorts and older systemic-therapy eras—report improved short- and long-term OS metrics for patients receiving P-HIPEC compared with surgery alone. These findings form much of the rationale for continued investigation and selective adoption in high-risk settings (57, 58). (2) Evidence showing no clear OS benefit (or uncertainty): Other analyses, including more recent studies and those incorporating Western practice patterns or more rigorous designs, have not consistently demonstrated an OS advantage (59). This inconsistency suggests that the efficacy of P-HIPEC may be highly context-dependent. The controversy over OS benefit may have multiple complex underlying reasons: ① Intrinsic heterogeneity of studies: Different studies have huge variations in patient selection criteria, quality of surgery, HIPEC regimens, and perioperative systemic chemotherapy regimens (3). Many early studies (from 20–30 years ago) were conducted before the popularization of modern high-efficiency systemic chemotherapy regimens (such as the FLOT regimen). The baseline prognosis of the control groups in these studies was inherently poorer, which may have exaggerated the relative survival benefit of P-HIPEC. ② Geographical and population differences: The vast majority of evidence currently comes from Asian countries, especially Japan, China, and South Korea (3). Considering the potential differences between Eastern and Western populations in the etiology, molecular subtypes, biological behavior of gastric cancer, and response to treatment, it has always been questioned whether the results from Asian studies can be directly extrapolated to Western populations. This is a major reason for initiating large-scale European clinical trials like GASTRICHIP—to validate its efficacy in a Western population. ③ Shift in metastasis patterns and impact of subsequent treatments: As a local therapy, P-HIPEC’s main role is to eradicate micrometastases in the peritoneal cavity to effectively prevent peritoneal metastasis. However, this treatment is powerless against pre-existing micrometastases in distant organs (such as the liver, lungs, and distant lymph nodes). Therefore, P-HIPEC may only modify the metastatic pattern by reducing peritoneal dissemination, while patients may ultimately still succumb to distant metastases. Furthermore, with advances in cancer treatment, even if a patient relapses, effective second- and third-line systemic treatments (including new chemotherapy drugs, targeted therapies, and immunotherapies) may prolong the post-recurrence survival time, thereby diluting or masking the direct impact of P-HIPEC on initial OS. The table below summarizes the efficacy data from some key RCTs and meta-analyses to visually present the current evidence and points of controversy in this field (**Table 2**).

**Table 2:** Efficacy results from key randomized controlled trials and meta-analyses of prophylactic HIPEC.

Study design/meta-analysis	Design	Numbers of patients	Peritoneal metastasis rate	DFS	OS	Main conclusion	Sources
Desiderio et al. (2023)	Meta (16 studies)	873/827	OR 0.22 [0.11, 0.47]	HR 0.80 [0.39, 1.65]	3-yr: OR 1.89 [1.17, 3.05], 5-yr: OR 1.87 [1.29, 2.71]	HIPEC improves 3-year and 5-year OS, reduces recurrence rate.	(58)
Xie et al. (2020)	RCT	68/62	Significantly lower (p=0.020)	Significantly improved (p=0.037)	Significantly improved (p=0.044)	P-HIPEC is beneficial for patients with cT4 stage gastric cancer	(59)
Gong et al. (2024)	Retrospective (PSM)	88/88	5.3% vs 17.3% (p=0.039)	HR 0.569 [0.362, 0.894]	HR 0.733 [0.447, 1.200]	HIPEC improves DFS, reduces PM, but does not improve OS.	(6)
Chen et al. (2024)	Meta (12 RCTs)	599/582	0.59 [0.50, 0.68] (Total metastasis)	RR 1.42 [1.07, 1.89]	3-yr: RR 0.63 [0.54, 0.73]	HIPEC improves survival, recurrence, and DFS.	(60)
Fan et al. (2023)	Meta (12 RCTs)	873/878	N/A	HR 0.78 [0.57, 1.07]	Gastric cancer subgroup: HR 0.49 [0.32, 0.76]	IPEC improves OS only in the gastric cancer subgroup.	(61)

Key meta-analyses evaluating prophylactic HIPEC in gastric cancer. Summary estimates and conclusions are reported as presented by the original meta-analyses; differences in included eras, systemic therapy backbones, and HIPEC protocols contribute to between-study heterogeneity. DFS, disease-free survival; HIPEC, hyperthermic intraperitoneal chemotherapy; HR, hazard ratio; OR, odds ratio; OS, overall survival; PM, peritoneal metastasis.

The value of P-HIPEC in controlling locoregional recurrence has been largely confirmed, but whether this can be translated into an ultimate survival benefit in the context of modern systemic therapy remains an unresolved core issue. Future large-scale, high-quality, and standardized RCTs are needed to provide a definitive answer.

## 7 Safety and complications of P-HIPEC

When considering the widespread application of a prophylactic treatment to a high-risk population, its safety is a consideration of equal importance to its efficacy. Historically, concerns about HIPEC potentially causing severe postoperative complications were a major obstacle to its widespread adoption. However, with the maturation of the technique and accumulation of experience, modern studies indicate that in skilled and experienced medical centers, the safety of P-HIPEC is acceptable.

### 7.1 Overall morbidity and mortality

Multiple studies and meta-analyses have shown that, compared to patients undergoing radical gastrectomy alone, the addition of P-HIPEC does not significantly increase the overall rate of severe postoperative complications (defined as Clavien-Dindo grade ≥ III) or surgery-related mortality. For example, an interim safety analysis of the ongoing PREVENT trial reported similar rates of serious adverse events (SAEs) in the HIPEC and control groups (35% and 30%, respectively), indicating that the addition of HIPEC did not introduce extra risk (50). This suggests that with strict patient selection and standardized procedures, P-HIPEC can be safely integrated into radical gastrectomy for gastric cancer.

### 7.2 Analysis of specific complications

Although the overall risk of complications is not significantly increased, P-HIPEC is associated with a higher incidence of certain specific toxic side effects related to chemotherapy drugs. ① Renal impairment: This is the most frequently reported and most significantly different specific complication of P-HIPEC. Multiple meta-analyses have consistently found that the incidence of postoperative renal insufficiency or acute kidney injury is significantly higher in the HIPEC group than in the control group, with ORs ranging from 2.44 to 3.94 (57, 58). This nephrotoxicity is mainly related to the intraperitoneal chemotherapy drugs used, especially drugs like cisplatin, which are metabolized by the kidneys and have known nephrotoxic effects (55). Therefore, strict preoperative assessment of renal function and active perioperative renal protective measures, such as hydration, are crucial for preventing such complications. ② Hematological toxicity: Neutropenia, thrombocytopenia, and anemia are common systemic toxicities caused by chemotherapy drugs entering the bloodstream. Although these complications occur after HIPEC, most meta-analyses have not found a statistically significant difference in the overall incidence of severe hematological toxicity between the HIPEC and control groups. This indicates that due to the peritoneal-plasma barrier, systemic absorption of intraperitoneally administered drugs is limited, and the resulting bone marrow suppression is mostly mild, manageable, and reversible. Studies have also pointed out that mitomycin C is an independent risk factor for severe hematological toxicity (62). ③ Gastrointestinal complications: (i) Anastomotic leak: This is a major concern for gastrointestinal surgeons. Early views suggested that hyperthermia and chemotherapy drugs might impair anastomotic healing. However, a large body of modern research data shows that because P-HIPEC does not involve extensive peritoneal stripping and cytoreductive surgery (CRS), the trauma to the intestine is relatively small, and thus the addition of HIPEC does not significantly increase the risk of anastomotic leak (63). This finding is important for promoting the application of P-HIPEC. (ii) Intestinal obstruction: The incidence of postoperative intestinal obstruction is usually not significantly different between the HIPEC and control groups (58). ④ Other complications: Some studies have reported a slight increase in pulmonary complications (such as pneumonia, pleural effusion) in the HIPEC group (one meta-analysis reported an OR of 6.03) (57), but this is not a consistent finding. As for complications like infection and fever, reports vary among studies, with some even suggesting that HIPEC may reduce the incidence of postoperative fever.

The safety of P-HIPEC is within an acceptable range. Its risks are mainly concentrated on foreseeable and manageable chemotherapy-related toxicities (especially nephrotoxicity), rather than unpredictable catastrophic surgical events. This requires the establishment of a multidisciplinary collaborative management model in clinical practice, with refined risk assessment and perioperative monitoring of patients.

## 8 Limitations and controversies of existing research

Although research on P-HIPEC in the treatment of gastric cancer has progressed, the current body of evidence still has numerous limitations and controversies. These issues are key to understanding the current clinical status and future direction of this technology.

### 8.1 Heterogeneity of evidence

This is the most fundamental and core challenge for P-HIPEC. The existing evidence, especially the primary studies on which meta-analyses rely, exhibits immense heterogeneity on multiple levels, which severely affects the reliability and generalizability of the conclusions. ① Varied study designs: There are significant differences among studies in patient inclusion criteria (e.g., stratification of CY1), choice of HIPEC drugs (MMC, cisplatin, oxaliplatin, etc.), technical protocols (temperature, duration, open/closed), surgical techniques (extent of D1/D2 lymph node dissection), and perioperative systemic chemotherapy regimens (3). The clinical significance of pooled effect sizes (such as OR or HR) obtained by simply mathematically combining the results of these disparately designed studies is necessarily questionable. ② Wide time span of studies: Many of the early key studies included in meta-analyses (such as the pioneering work by Koga et al.) were completed in the 1980s and 1990s (64). In that era, the standardization of gastric cancer surgery, the level of perioperative management, and the effectiveness of systemic chemotherapy were far inferior to today’s standards. In particular, highly effective perioperative chemotherapy regimens like FLOT were not available. Therefore, the prognostic baseline of the control groups in these studies (usually surgery alone) was much poorer than that of control groups under modern standards, which may have exaggerated the relative survival benefit of P-HIPEC.

### 8.2 Geographical limitation of evidence

The vast majority of high-quality evidence supporting the effectiveness of P-HIPEC comes from Asian countries, particularly China, Japan, and South Korea (3). This pronounced geographical bias raises a critical question: can the positive results observed in Asian populations be directly applied to Western populations? There may be differences between Eastern and Western populations in the epidemiological characteristics, main pathological types, molecular subtype profiles (e.g., proportions of EBV-associated, MSI-H, GS, CIN), and response to chemotherapy for gastric cancer. Therefore, using Asian experience as the basis for global guidelines is highly controversial in the absence of evidence from large, prospective RCTs in Western populations. This is also the core rationale driving European trials such as GASTRICHIP (3). In addition, differences in perioperative standards (extent of lymphadenectomy, perioperative chemotherapy uptake, and postoperative surveillance) may modify both baseline peritoneal recurrence risk and the absolute benefit achievable with P-HIPEC. Therefore, subgroup reporting by region/era and chemotherapy backbone (FLOT-era vs non-FLOT-era) should be prioritized, and conclusions derived from predominantly Eastern, pre-FLOT datasets should be framed as hypothesis-generating for contemporary Western practice.

### 8.3 Uncertainty of overall survival benefit

As mentioned above, the uncertainty surrounding overall survival benefits remains the central point of contention. Current studies have shifted from simply comparing the efficacy of HIPEC versus surgery alone to exploring whether prophylactic HIPEC can provide additional survival benefits when combined with modern, highly effective perioperative systemic chemotherapy regimens (such as FLOT). Many oncologists remain skeptical, arguing that the local control effects of prophylactic HIPEC may be overshadowed or weakened by the potent systemic effects of contemporary chemotherapy. Addressing this issue is a primary objective of clinical trials such as PREVENT.

### 8.4 Lack of technical standardization and long-term quality of life data

Currently, there is no globally recognized, standardized technical operating procedure for P-HIPEC. The optimal chemotherapy drugs, the most appropriate drug dosages, the ideal perfusion temperature and duration, and the pros and cons of open versus closed techniques are all without definitive conclusions. This “kaleidoscopic” clinical practice not only makes it difficult to compare study results but also poses significant obstacles to the dissemination and quality control of the technology. Furthermore, most studies focus on survival and recurrence rates as endpoints, while high-quality data on the long-term impact of P-HIPEC on patients’ Quality of Life (QoL) are relatively scarce. This is an important gap that future research needs to address.

From a patient-centered perspective, future trials should incorporate validated patient-reported outcome measures (PROMs) alongside oncologic endpoints to clarify the net clinical benefit of P-HIPEC. A pragmatic framework would include (i) early postoperative recovery domains (pain, bowel function, fatigue), (ii) longer-term gastrointestinal function and nutritional status, and (iii) global quality of life at standardized timepoints (e.g., baseline, 3, 6, 12 months, then annually). Reporting PROM completion rates and minimally important differences will be essential to interpret whether reduced peritoneal recurrence translates into meaningful survivorship benefit.

### 8.5 Practical standardization checklist

To improve interpretability and enable cross-study comparison, future reports of P-HIPEC in gastric cancer should, at minimum, specify:

1. Intended setting: prophylactic (P0/CY0) vs early-therapeutic (P0/CY1) vs macroscopic PM (therapeutic CRS/HIPEC).
2. Baseline systemic regimen: perioperative chemotherapy details (e.g., FLOT vs non-FLOT), number of cycles, and compliance.
3. Surgical quality: extent of gastrectomy, lymphadenectomy (D1/D2), R0/R1, and standardized postoperative care.
4. HIPEC technique: open vs closed, perfusion system, target intraperitoneal temperature (range and monitoring sites), flow rate, and duration with rationale.
5. Drug regimen: agent(s), dose calculation (mg/m² vs fixed), carrier solution, timing relative to anastomosis, and any nephroprotection strategy (for cisplatin).
6. Safety definitions: complication grading (e.g., Clavien–Dindo), toxicity criteria (e.g., CTCAE), and explicit definitions for renal/pulmonary events.
7. Outcomes: peritoneal recurrence, DFS, OS, and patient-reported outcomes (when available), with prespecified follow-up schedules.

## 9 Future prospects

Facing the current controversies and shortcomings, P-HIPEC is at a critical crossroads. Future development will focus on clarifying its precise role in the modern multidisciplinary comprehensive treatment of gastric cancer through high-quality clinical trial evidence, technological innovation, and precision strategies.

A series of rigorously designed, large-scale Phase III RCTs are currently underway. Their results are expected to answer many of the long-standing controversies in the field and may guide future clinical practice guidelines. ① GASTRICHIP (NCT01882933): This French-led, multicenter European RCT aims to directly validate the efficacy of Asian experience in a Western population. The trial randomly compares the efficacy of oxaliplatin HIPEC versus no HIPEC after D2 radical surgery, with 5-year OS as the primary endpoint. Its results will be persuasive (47). ② PREVENT (FLOT9, NCT04447352): This German-led RCT specifically targets patients with diffuse-type gastric cancer, who have the highest risk of peritoneal metastasis. In the context of the current optimal perioperative FLOT chemotherapy, it compares the efficacy of surgery combined with cisplatin HIPEC versus surgery alone, with DFS as the primary endpoint. The design of this trial addresses the question of the additional value of P-HIPEC on top of the most potent modern systemic therapy (50). ③ CHIMERA (NCT04597294): This Polish-led RCT has made a bold innovation in treatment timing. It explores a unique treatment model of neoadjuvant (preoperative) laparoscopic HIPEC combined with perioperative FLOT. The theoretical basis is that by using minimally invasive laparoscopic HIPEC to clear micrometastases in the peritoneal cavity before systemic chemotherapy and radical surgery, it may be possible to block the metastatic process earlier (51). The designs of these trials are distinctive, exploring the value of P-HIPEC from different dimensions such as population, chemotherapy background, drug selection, and treatment timing. Even if their final results differ, they will greatly enrich our understanding of P-HIPEC and promote its application from a “one-size-fits-all” approach to a more refined and individualized one.

New technologies are beginning to be applied in the clinic: ① New drug delivery systems: To overcome the disadvantage of uneven drug distribution in traditional closed-method HIPEC, new technologies are emerging. For example, “Pressurized Intraperitoneal Aerosol Chemotherapy (PIPAC)” uses laparoscopy to aerosolize chemotherapy drugs, utilizing pressure to distribute them evenly over the peritoneal surface. Additionally, some new HIPEC systems generate turbulence by injecting CO2 gas into the peritoneal cavity to improve the distribution of liquid drugs (65). ② Integration of minimally invasive techniques: Combining the precise operation and minimally invasive advantages of “Robotic Surgery” with HIPEC is expected to further reduce surgical trauma and postoperative complications while ensuring oncological radicality, thereby accelerating patient recovery (66). ③ New drugs and combination therapies: Future research will no longer be limited to traditional chemotherapy drugs but may explore the application of novel drugs in HIPEC. Even more anticipated is the combination of HIPEC, an efficient local therapy, with systemic targeted therapies or immune checkpoint inhibitors to achieve a three-dimensional attack on both local micro-lesions and systemic micrometastases.

At the level of patient selection, the future goal is to achieve true individualized treatment, providing P-HIPEC to patients most likely to benefit while avoiding unnecessary treatment for non-responsive or high-risk patients. Future research will focus on finding reliable molecular biomarkers that can predict the risk of peritoneal metastasis and the efficacy of P-HIPEC. These biomarkers may be derived from tumor tissue (e.g., specific gene mutations, EMT-related gene expression profiles), peritoneal washings, or by detecting circulating tumor DNA (ctDNA) or specific molecules in exosomes (e.g., miRNAs) in peripheral blood through liquid biopsy techniques (67). This would allow for the precise identification of the best candidates for P-HIPEC preoperatively or intraoperatively.

P-HIPEC should not be regarded as an independent treatment modality but should be integrated into the entire perioperative multidisciplinary treatment system for gastric cancer. Future research needs to further optimize the integration model of P-HIPEC with modern systemic chemotherapy regimens (such as FLOT), including the optimal timing and sequence of administration, to maximize their synergistic anti-tumor effects and minimize the risk of overlapping toxicities (50, 51).

### 9.1 Clinical practice implications

1. Who may benefit most: Patients with non-metastatic but high-risk disease (e.g., serosal involvement pT4a, bulky nodal disease, diffuse/mixed Lauren type) undergoing high-quality curative gastrectomy with appropriate perioperative systemic therapy.
2. Where evidence is strongest: Consistent reduction in peritoneal recurrence and improvement in disease-free survival in many studies, predominantly from Asian cohorts and pre-FLOT eras.
3. Where evidence is uncertain: Overall survival benefit remains inconsistent across meta-analyses and populations; generalizability to Western/FLOT-era practice is not yet definitive.
4. Key risks to discuss: Drug-specific toxicities (notably cisplatin-associated acute kidney injury), perioperative morbidity, and the impact of protocol variability (drug/dose/temperature/duration, open vs closed technique).
5. MDT discussion points: Intended setting (P0/CY0 prophylaxis vs P0/CY1 early-therapeutic), expected absolute recurrence risk, institutional HIPEC expertise/quality assurance, patient comorbidity/renal reserve, and availability of clinical trials.

## 10 Conclusion

Prophylactic Hyperthermic Intraperitoneal Chemotherapy (P-HIPEC), as a local therapeutic strategy aimed at reducing the risk of peritoneal metastasis after radical gastrectomy for gastric cancer, has had its value in controlling locoregional disease well-affirmed. A substantial body of evidence clearly indicates that for high-risk gastric cancer patients, intraoperative P-HIPEC can significantly reduce the risk of postoperative peritoneal metastasis and effectively prolong disease-free survival, which forms its most solid basis for clinical application. However, despite its significant effect in preventing metastasis, whether it can ultimately translate into an improvement in overall survival remains the biggest controversy in the field. The conflict in existing evidence mainly stems from the high heterogeneity of studies, the geographical limitation of evidence (mainly from Asia), and the potential confounding effect of modern high-efficiency systemic chemotherapy regimens on overall patient survival. In terms of safety, P-HIPEC is considered acceptable in experienced multidisciplinary treatment centers. It does not significantly increase the risk of severe surgery-related complications such as anastomotic leak, but its specific renal toxicity is a definite risk that requires high vigilance and management. The future direction for P-HIPEC lies in precision, standardization, and integration. The focus of the academic community has shifted from “whether it is effective” to “for whom it is effective” and “how to be most effective.” The results of large-scale Phase III clinical trials, represented by GASTRICHIP and PREVENT, will provide higher-quality evidence for the application of P-HIPEC in the context of modern therapy. Exploring new biomarkers to achieve individualized screening and deeply integrating it with systemic treatments such as targeted and immune therapies will be the core driving force for advancing the field. The value of P-HIPEC is not as a universal standard treatment, but as an indispensable part of individualized, multidisciplinary comprehensive therapy, offering a significant opportunity for carefully selected high-risk patients to reduce their risk of metastasis and improve their prognosis.
